# Jump-Starting a Cellular World: Investigating the Origin of Life, from Soup to Networks

**DOI:** 10.1371/journal.pbio.0030396

**Published:** 2005-11-15

**Authors:** Richard Robinson

## Abstract

If we agree that complex life evolved ultimately from single-celled organisms, how do we explain the origins of the cell itself?

A physicist, a chemist, and a mathematician are stranded on a desert isle, when a can of food washes up on the beach. The three starving scientists suggest, in turn, how to open the can and ease their hunger. The physicist suggests they hurl it upon the rocks to split it open, but this fails. The chemist proposes they soak it in the sea and let the salt water eat away at the metal; again, no luck. They turn in desperation to the mathematician, who begins, “Assume we have a can opener….”

When discussing the evolution of life, biologists can often sound a bit like that mathematician. Beginning with a single cell, Darwinian evolution provides a simple, robust, and powerful algorithm for deriving all the astonishing richness of life, from bacteria to brains. Natural selection and other evolutionary forces, acting on surplus populations of replicating cells and multicellular organisms, lead inevitably to evolution and adaptation. Give biologists a cell, and they'll give you the world. But beyond assuming the first cell must have somehow come into existence, how do biologists explain its emergence from the prebiotic world four billion years ago?

The short answer is that they can't, yet. But this question may be a little closer to being answered as new money enters the field, and two new discoveries provide support for two competing models of prebiotic evolution.

While the past half century has seen an explosion of knowledge about the evolution of life after it began, there has been relatively little progress in the past half century on how it began—the so-called origin question. In part, the problem is financial: research money has flooded many other areas in biology, but remains in short supply in this one. “The funding is a big part of it,” says Jack Szostak, a Howard Hughes investigator and Professor of Genetics at Harvard Medical School. As a result, there is a shortage of researchers willing to commit their professional careers to finding out how life began. “This is a risky field to be involved with. The problems are hard. You can train students, but there may not be jobs waiting for them afterwards.” To that end, Harvard University recently announced a plan to fund origin-of-life research to the tune of one million dollars per year, which Szostak says is a good start.

Beyond assuming the first cell must have somehow come into existence, how do biologists explain its emergence from the prebiotic world four billion years ago?

But finding the answer to the origin question will require not only money but also progress in understanding how the most basic of biological molecules were put together before life began, how they became organized and self-sustaining, and how they developed into the membrane-bound cells that are our ancestors. Scientists have come a long way from the early days of supposing that all this would inevitably arise in the “prebiotic soup” of the ancient oceans; indeed, evidence eventually argued against such a soup, and the concept was largely discarded as the field progressed. But significant problems persist with each of the two competing models that have arisen—usually called “genes first” and “metabolism first”—and neither has emerged as a robust and obvious favorite. Now, two papers published in mid-2005 offer each camp some encouragement.

## The Origin of Origin-of-Life Experiments

Some 50 years ago, Stanley Miller, then a graduate student at the University of Chicago and now in the Department of Chemistry at University of California at San Diego, got the field of origins research started with a bang—literally. He passed high-voltage electric sparks—a stand-in for lightning—through a gaseous mixture of water, methane, hydrogen, and ammonia, thought to be the major constituents of the ancient atmosphere. The liquid in the reaction flask eventually became a bouillon-like mix of amino acids and other small organic molecules. Miller's results predicted that, over time, the early oceans would have become a rich prebiotic soup, replete with amino acids, nucleic acids, and sugars. His results implied it was only a matter of time before these building blocks combined to form complex polymers and ultimately a replicating cell.

In the beginning, according to the so-called genes-first camp, was a single RNA molecule, both code and catalyst. Such a “replicase” would have catalyzed its own replication, and also provided the template on which the copy was made.

“The initial Miller experiment was earth-shaking,” says Harold Morowitz, Professor of Biology at George Mason University, and a long-time theorist and researcher in this area. The suggestion that random chemistry could produce the molecules of life “held the field for a long time.” But later calculations appeared to show that the early atmosphere contained much more carbon dioxide and much less hydrogen than Miller's model required, and correcting these concentrations cast doubt on the likelihood that complex molecules would form in abundance Where, then, might organic precursors have come from? There is some, albeit scant, evidence for their arrival on comets colliding with the earth, but there is little enthusiasm for this as a solution. Finally, there is no geologic evidence, in either sediments or metamorphic rocks, that such a soup ever existed.

## An RNA World Needs Nucleotides

In the early 1980s, just as Miller-type chemistry was falling out of favor, RNA emerged as the rising star of origin-of-life research, based on a startling discovery. Up to this point, evolution appeared to have a severe chicken-and-egg problem: information-bearing DNA codes for protein, but catalytic proteins are essential to make DNA. That the two could have arisen independently but still work in concert seemed highly unlikely. But RNA, which was well-known in its role as temporary information carrier, also turned out to be catalytic. Indeed, a host of functions in modern cells that were once thought to be the province of proteins are instead supervised by catalytic RNA. It is only a small intellectual leap from here back to an “RNA world,” in which RNA, not DNA, is the molecule of heredity, and RNA, not protein, is the catalytic engine of the cell. In the beginning, according to the so-called genes-first camp, was a single RNA molecule, both code and catalyst. Such a “replicase” would have catalyzed its own replication, and also provided the template on which the copy was made.

Work by Jack Szostak of Harvard University has lent support to this elegant model. He has shown that certain catalytic RNAs can, indeed, join smaller RNA sequences together, hinting at the potential for self-replication. Given the right starting conditions, such a self-replicating RNA might increase its number at the expense of the “lifeless” ones surrounding it. Successive rounds of copying, with minor mutations, could lead the original replicator to acquire new abilities. Life, Szostak speculates, “starts simple, beginning with one gene, probably a replicase, and accretes additional functionality over time.”

It is a highly appealing concept, and has driven a great deal of good research. But how would the original replicase arise? James Ferris, Professor of Chemistry at Rensselaer Polytechnic Institute, has discovered that on the surface of montmorillonite, a common clay, activated RNA nucleotides—the monomeric building blocks of the RNA polymer—will spontaneously link together to form longer chains. While the sequences of these products are entirely random, Szostak has shown that within such a random pool of RNAs, some are likely to be catalytic. Szostak has also recently shown that replicating RNAs inside a lipid membrane vesicle cause the vesicle to grow, mimicking behavior of actual cells.

**Box 1. How Did Life Become Handed?**

To date, none of the models have proposed a solution to one of the more vexing origin problems: chirality. Three-dimensional molecules such as sugars and amino acids can exist in two mirror-image forms, like left and right hands (chiros is Greek for hand). Any nonbiological synthesis of such molecules, as would have occurred before life arose, produces equal amounts of each type. Nonetheless, modern cells use exclusively left-handed amino acids and right-handed ribose sugars, and interference from the wrong kind shuts down biological reactions. How could chiral life arise in the presence of so much interference?

“It's a serious problem,” Orgel admits, “but not an overwhelmingly serious one.” Orgel suggests that one of several possible solutions may be chance, a “frozen accident” that brought together, and kept together, molecules of the right chirality. Such an accident is perhaps not so unlikely, says Martin, who calculates that a mixture of every possible left- and right-handed combination of a 25–amino acid peptide (amino acid chain) would weigh 25 kilograms. “Any smaller sample is imperfect,” he says.

Martin also points out the problem may be a bit easier than it seems, since the chirality of a molecule such as a sugar is usually maintained as that molecule wends its way through a metabolic pathway. An enzyme at the head of that pathway could act as a “filter,” allowing only those molecules of the correct chirality to enter, thus fixing chirality for that pathway and others that branch off of it. Exactly this feature is seen in the central metabolic pathway for sugar formation found in all cells.

But working back even further, where do the nucleotides come from to form these chains? Here we come up against the “can opener” problem on the molecular level. “The biggest concern about the RNA world is that there has been no convincing prebiotic creation of the activated monomers” in any plausible prebiotic world, says Ferris. Despite years of experiments with dozens of different strategies, no one has figured out how to make this most essential of starting ingredients for an RNA world. “There is a growing realization that we may need to look beyond RNA,” Szostak says, to molecules whose chemistry is a bit more tractable, such as a peptide nucleic acid (PNA), a synthetic amino acid–nucleotide hybrid. These original replicators might then have given way to RNA, says Leslie Orgel, senior fellow and research professor at the Salk Institute of Biological Studies.

The case for PNA is weak, though. While modern cells still bear traces of a catalytic RNA world within them, “there is absolutely nothing that I know of to suggest there is evidence for PNA or other such molecules in present cells,” says Orgel. If they ever contributed to the development of life, all traces of their existence appear to have been wiped clean.

Whether the original replicator was RNA or PNA or some other molecule, any genes-first model relies on an abundance of building blocks in the environment, a requirement that seems to depend on the discounted idea of Miller's prebiotic soup. But in June of 2005, the prebiotic soup got a new lease on life. New calculations appear to show that there was considerably more hydrogen in the early atmosphere than once thought. “This could resurrect Miller's chemistry,” says Orgel. Nonetheless, “there is still an enormous way to go” to get the full set of RNA precursor molecules.

## Metabolism More Ancient than Replication?

In 1988, even while the RNA world was enjoying its intellectual honeymoon, a German biochemist and patent attorney, Günther Wächtershäuser, proposed a radical alternative theory of the origin of life based on, of all things, fool's gold. Iron disulfide—pyrite or fool's gold—can catalyze a variety of crucial biochemical reactions. There are iron sulfide or iron–nickel sulfide clusters at the heart of several ancient and vital enzymes in use in all cells today. Wächtershäuser proposed that the earliest living system was not a nucleotide-based replicator but a mineral-based metabolizer, converting simple and abundant inorganic compounds—carbon dioxide, hydrogen sulfide—into more complex organic ones on the surface of a pyrite crystal, probably at deep-sea hydrothermal vents.

This metabolism-first model has a strong champion in Harold Morowitz, who paints the two major models for life's origin as “heaven and hell.” Miller-type scenarios, including the genes-first model, rely on a wealth of precursors raining down from above. The standard model of the RNA world, he says, “requires an environment that is impossibly improbable.” The alternative is a much smaller set of molecules, at much higher concentrations, bubbling up from below. “I really like theories that go from simplicity to complexity,” Morowitz says.

The possibility that metabolism first began at hydrothermal vents has been advanced most recently by Michael Russell, Research Professor of Geology at the Scottish Universities Environmental Research Centre in Glasgow, and William Martin, Professor at the University of Düsseldorf. Russell and Martin propose that life's metabolism developed not on a two-dimensional pyrite surface but within tiny cavities lined with iron monosulfide, through which percolated an energy-rich mix of hydrogen and carbon dioxide dissolved in seawater.

The standard model of the RNA world...“requires an environment that is impossibly improbable.” The alternative is a much smaller set of molecules, at much higher concentrations, bubbling up from below.

**Figure 1:**
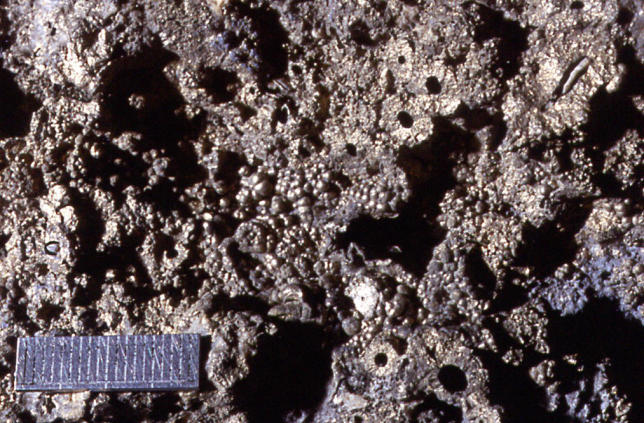
Tynagh Chimneys A view from above a chimney field, showing the chimneys (round black circles) and bubbles, which contain chambers. The object placed for scale is two centimeters across. These fossil chimneys were formed well after life's origin, but may be similar to those in which, according to one hypothesis, metabolism first began

**Figure 2:**
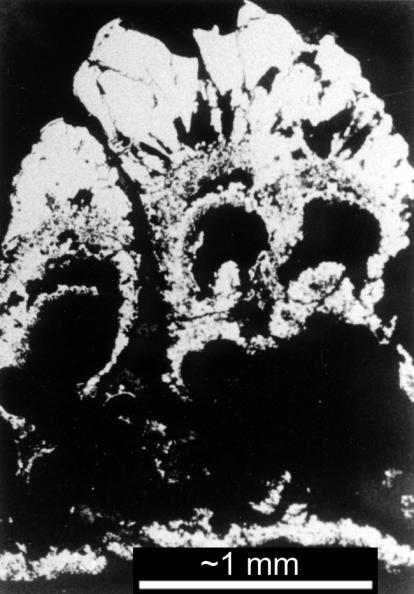
Botyroidal Cross-Section A cross-section through an iron sulfide deposit shows the small chambers within. One hypothesis of life's origin suggests that in such chambers metabolism first began, as hydrogen and carbon dioxide bubbled through and reacted to form simple organic compounds

In the early 1980s, Russell discovered fossil fields of iron sulfide “chimneys,” formed on the ocean floor 350 million years ago (Figures 1 and 2). Unlike the more famous and much larger “black smokers”—deep-sea hydrothermal vents found in mid-ocean ridges that spew hot, mineral-rich water out of their sulfide chimneys—each of Russell's chimneys is no more than ten centimeters high and little more than a centimeter across. The entire field covers tens of square meters, and is composed, in part, of many thousands of millimeter-sized cavities, formed by outgassing of hydrogen and carbon dioxide, which bubbled up through cracks in the crust. While the particular structures Russell discovered formed well after life's origins, similar ones almost certainly existed in the prebiotic ocean, Russell says. Each would have remained stable over the course of thousands of years, a little experimental reaction vessel at the bottom of the sea.

And unlike the 400 °C water spewing out of a black smoker, the water flowing up through these vents was much cooler, not much more than 100 °C. Outside the chamber, the ocean would have been much cooler still, and more acidic and more oxidized than the solution within, creating a set of strong temperature and electrochemical gradients across the microscopically porous surface of the chamber. “Life loves to live at the gradients,” says Russell.

The slow trickle of hydrogen and carbon dioxide through such chambers and across the iron sulfide catalyst promotes formation of acetate, according to Russell and Martin. Acetate is a key intermediate in virtually all biosynthetic pathways, and in modern cells, enters these reactions tethered to sulfur. In modern bacteria, the two enzymes that make acetate depend on a catalytic core of iron, nickel, and sulfur, arranged almost exactly as they are in the free mineral itself. “In other words,” Russell and Martin have written, these enzymatic metal clusters “are not inventions of the biological world, rather they are mimics of minerals that are indisputably older, and which themselves have catalytic activity in the absence of protein” [1].

These chambers also suggest a radical solution to a heretofore stubborn problem, one with no other obvious resolution. Modern cells without nuclei are grouped into two domains, the Eubacteria and the Archaebacteria. These ancient lineages share the same energy metabolism and the same genetic code, presumably reflecting a single common ancestor. But they differ profoundly in how they synthesize the lipids in their membranes. One explanation, which Martin dismisses, is that the common ancestor had a membrane, probably similar to the eubacterial structure, and the archaebacterial ancestor “had to completely reinvent its cell wall chemistry.” Such proposals are “completely decoupled from microbial physiology,” he says. The alternative favored by Russell and Martin is that these lipid differences reflect a divergence in the two lines after the last common ancestor already had its carbon biochemistry and genetic code intact, but before the development of lipid membranes. The chambers served as the original cell compartment, and were only replaced by lipids after the eubacterial and archaebacterial lines split.

This metabolism-first model is not an alternative to life based on RNA...But it does propose that geology at hydrothermal vents provided the structure in which life emerged

Russell and Martin's model also provides a solution to another thorny issue in jump-starting life, that of concentration. An essential feature of all cells is their ability to maintain high concentrations of materials that are in short supply in the world around them. In the absence of a cell membrane, how did proto-life forms collect raw materials, and prevent products from dissipating into the vastness of the environment around them? Russell's chambers solve this problem in essentially the same way modern cells do, with an external boundary that is permeable to small reactant molecules, but much less so to larger product ones. In Russell's and Martin's scenario, then, the stable metabolism that developed within these chambers eventually gave rise to a genetic system, probably dependent on RNA, which encoded simple proteins, probably through direct accretion of RNA and amino acids on the surface of a mineral catalyst. Finally, these proto-organisms developed membranes, completing their evolution into recognizable cells.

This metabolism-first model is not an alternative to life based on RNA. “We can't work without an RNA world either,” says Martin. But it does propose that geology at hydrothermal vents provided the structure in which life emerged, and suggests that understanding prebiotic organic chemistry at these vents may provide the key to understanding the emergence of life from nonlife.

## Self-Organizing Metabolic Networks

While not necessarily convinced of the details of this proposal, Morowitz applauds the focus on bacterial physiology as a guide to understanding early life. “Metabolism recapitulates biogenesis,” he proposes.

But for Morowitz, the most exciting development in the metabolism-first camp, “the really new idea,” is that small organic molecules, such as amino acids, can catalyze the formation of other small organic molecules, such as nucleic acids. “This has emerged only in the last two years,” he says. This view has found strong support from a new finding published in the journal *Chemistry* in August 2005, which indicates that single amino acids can catalyze the creation of sugars from simple starting materials with enzyme-like specificity.

“What has emerged is a very strong self-organizing principle,” says Morowitz. In this view, while iron sulfide may have been the original catalyst, it did not remain the only one for long. As products of the original reactions catalyzed new reactions, metabolic networks quickly arose. Feedback loops developed when two molecules regulated one another's synthesis. “The system can piggyback its way upward,” he says.

While the study of such networks is still in its infancy, Morowitz suggests they hold the key to a host of knotty problems, including that of RNA synthesis. “It's not a problem in this network point of view. Very early on you get the precursor compounds,” while formation of the complete nucleotide arises later. “Even today this is the core network of biochemistry.”

It is still unclear how, or whether, these competing models will fit together, and whether they will lead to a robust scenario for life's origin. Indeed, all may eventually prove wrong, and the real solution may lie hidden in some discovery yet to be made. Whatever the difficulties, says Morowitz, the allure of the field lies in its potential to answer the biggest question of them all. “You're not going to make drugs or better agriculture. You're going to make a philosophical impact.” Szostak agrees: “These are the big questions. Anybody who thinks has to be grabbed by these.”

## Abbreviation

PNA: peptide nucleic acid
